# Thermophilic Hemicellulases Secreted by Microbial Consortia Selected from an Anaerobic Digester

**DOI:** 10.3390/ijms25189887

**Published:** 2024-09-13

**Authors:** Luca Bombardi, Marco Orlando, Martina Aulitto, Salvatore Fusco

**Affiliations:** 1 Biochemistry and Industrial Biotechnology (BIB) Laboratory, Department of Biotechnology, University of Verona, 37134 Verona, Italy; 2 Department of Biotechnology and Biosciences, University of Milano Bicocca, 20126 Milano, Italy; 3 Department of Biology, University of Naples Federico II, 80126 Naples, Italy

**Keywords:** microbial consortia, lignocellulose biomass, carbohydrate-active enzymes, phylogenetic analyses, xylanases

## Abstract

The rise of agro-industrial activities over recent decades has exponentially increased lignocellulose biomasses (LCB) production. LCB serves as a cost-effective source for fermentable sugars and other renewable chemicals. This study explores the use of microbial consortia, particularly thermophilic consortia, for LCB deconstruction. Thermophiles produce stable enzymes that retain activity under industrial conditions, presenting a promising approach for LCB conversion. This research focused on two microbial consortia (i.e., microbiomes) that were analyzed for enzyme production using a cheap medium, i.e., a mixture of spent mushroom substrate (SMS) and digestate. The secreted xylanolytic enzymes were characterized in terms of temperature and pH optima, thermal stability, and hydrolysis products from LCB-derived polysaccharides. These enzymes showed optimal activity aligning with common biorefinery conditions and outperformed a formulated enzyme mixture in thermostability tests in the digestate. Phylogenetic and genomic analyses highlighted the genetic diversity and metabolic potential of these microbiomes. *Bacillus licheniformis* was identified as a key species, with two distinct strains contributing to enzyme production. The presence of specific glycoside hydrolases involved in the cellulose and hemicellulose degradation underscores these consortia’s capacity for efficient LCB conversion. These findings highlight the potential of thermophilic microbiomes, isolated from an industrial environment, as a robust source of robust enzymes, paving the way for more sustainable and cost-effective bioconversion processes in biofuel and biochemical production and other biotechnological applications.

## 1. Introduction

The increase in agro-industrial activities over the past decades has led to the production of tons of discarded lignocellulose biomasses (LCB). LCB derived primarily from plant materials represents one of the most abundant renewable resources available for bioenergy and bio-based products. The main sources of cellulosic biomass include agricultural residues (such as straw, corn stover, and sugarcane bagasse), forestry residues (including wood chips, sawdust, and forest thinning), and dedicated energy crops (like switchgrass, miscanthus, and short-rotation coppice species). Recent statistics indicate that the global annual production of lignocellulosic biomass is approximately 181.5 billion tons, of which only 8.2 billion tons are utilized across various application areas [1]. LCB are abundant and inexpensive feedstocks that serve mainly as a source of fermentable sugars, chemicals, and other renewable products [2]. Typically, LCB hydrolysis at industrial settings involves harsh methodologies, such as acid hydrolysis or thermal shock, that imply a high cost of solvents and contribute to the production of toxic compounds [3,4], which in turn inhibit subsequent microbial fermentation [5]. For example, acid hydrolysis typically involves high temperatures (160–230 °C) and pressures (~10 atm). The acid concentration in the concentrated-acid hydrolysis process ranges from 10 to 30%, necessitating the use of corrosion-resistant reactors, which significantly increases the cost of pretreatment [4]. The use of supercritical fluids can be a valid alternative, as they guarantee a better biomass conversion into valuable products [6], but most of them are non-biodegradable and contribute to environmental pollution [7]. For this reason, the use of microbial consortia (i.e., microbiomes) for the deconstruction of biomasses has started to be considered as a suitable alternative approach, due to their capability to efficiently transform LCB into organic products [2].

In this framework, thermophiles have gained attention in recent decades as a source of industrially relevant thermostable enzymes acting on LCB [8,9]. These microbes can thrive at 41–122 °C with optimal growth temperatures ranging between 60 and 108 °C [8]. Unlike mesophilic enzymes, those produced by thermophiles exhibit a higher transition temperature (T_m_) of unfolding and a longer half-life at high temperatures, also in the presence of organic solvents [10]. Consequently, thermophilic and thermostable enzymes allow a good compromise to be obtained between activity in harsh industrial conditions (extreme pH, high temperature, presence of organic solvents, etc.) and experimental reproducibility [11]. LCB deconstruction necessitates the synergistic action of different enzymes to convert their polysaccharides into fermentable sugars. To obtain efficient saccharification, it is necessary to consider the chemical composition of the original feedstock and the pretreatment method [12,13]. Currently, commercial cocktails primarily rely on the mesophilic fungal secretomes from *Trichoderma reesei*, which exhibit optimal activity at temperatures between 45 and 55 °C, but rapidly inactivate over time. Thus, research has focused attention on the discovery of new thermostable enzymes that can enhance the saccharification properties of commercial cocktails [11]. For instance, Bernardi et al. (2019) [14] reported that the supplement of Celluclast^®^ 1.5 L with a thermostable endo-1,4-β-glucanase (Af-EGL7) from *Aspergillus fumigatus* EGL7 results in better degradation and an increase in released reducing sugars from corncob and rice straw (128% and 80%, respectively) [14]. Similarly, the yield of glucose released from steam-exploded sugarcane bagasse hydrolysis increased after the addition of a cocktail of recombinant hemicellulases from *Humicola grisea* var *thermoidea* and *Penicillium pupurogenum* to the commercial cellulase Accelerase 1500 [15]. In the landscape of enzymes involved in the bioconversion of LCB to sugars, referred to as carbohydrate active enzymes (CAZymes) [16], xylanases have gained growing attention due to their versatility in industrial applications. In the pulp and paper industry, xylanases are used during the pretreatment of pulps before bleaching and enhance the extraction of lignin [17]. Additionally, the discovery of novel xylanases, used either alone or in formulated enzymatic cocktails, can provide interesting new perspectives towards more efficient LCB deconstruction [18].

To facilitate the identification of microorganisms and enzymes involved in saccharification processes, specific enrichment cultures can be set up by formulating culture media with LCB as the sole carbon source. Thermophilic anaerobic digestion has been widely explored as a bacterial inoculum for LCB degradation and improved biomethane production [19]. The use of thermophiles provides several benefits, including improved kinetics, efficient degradation of organic matter, and economic and environmental sustainability. Ho et al. (2013) [20] reported that the growth rate of thermophilic methanogens is 2 to 3 times higher than that of the mesophilic methanogens, with reduced hydraulic retention time. In addition, the process can lead to enhanced digestate quality and higher rates of pathogen removal as compared to mesophilic anaerobic digestion [21]. The use of thermotolerant cellulolytic bacterial consortia containing *Clostridiaceae* isolated from digestate sludge and *Lachnospiraceae* isolated from cow manure led to a 3–11-fold increase in biogas production from oil palm empty fruit bunches [22]. The development of microbe-based strategies is still challenging due to a lack of information about the active microbial community and microbial indicators, which are dependent on different process conditions [23]. Moreover, there are studies about the presence of thermophilic communities in mesophilic anaerobic fermenters, but it remains almost unknown how physiologically and metabolically active these bacteria are [24]. Therefore, this study aimed to investigate the lignocellulose-degrading potential of thermophilic microbiomes isolated from a local anaerobic digester (i.e., a biogas plant). Microbiomes were grown on selective substrates, i.e., carboxymethylcellulose (CMC) and xylan (XYL), and tested for their ability to produce hydrolytic enzymes in a cheap medium i.e., a mixture of spent mushroom substrate (SMS) and digestate. Secreted enzymes (i.e., secretomes) were characterized in terms of temperature and pH optima, thermal stability (including shelf-life at 4 °C), and types of products released upon hydrolysis of pure and complex hemicellulose-derived polymers. Finally, phylogenetic and CAZome analyses are provided to unravel the genomic complexity and the biotechnological potential of these microbiomes.

## 2. Results

### 2.1. Thermophilic Microbial Consortia Secrete Robust Xylanases

**Figure 1:**
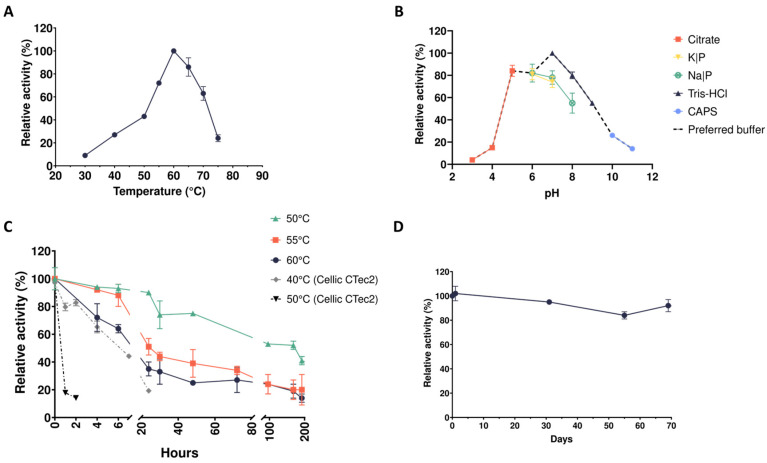
Biochemical characterization of xylanase activities in the secretome from the microbiome CMC-50. (**A**) Temperature optimum; (**B**) pH optimum; (**C**) thermostability; (**D**) storage stability.

As reported in our previous work, five microbiomes were isolated from enrichment cultures carried out at three different temperatures (37, 50, and 70 °C) on three carbon sources, i.e., spent mushroom substrate (SMS), carboxymethyl cellulose (CMC), and xylan (XYL) [25]. In this study, sterile SMS and the digestate (obtained from an anaerobic digestor) were mixed to proliferate thermophilic microbiomes and to induce the secretion of lignocellulolytic enzymes. Azo-xylan assays were performed on cell-free supernatants (CFS) derived from microbiomes selected at 50 °C (i.e., CMC-50 and XYL-50) and revealed the presence of xylanolytic enzymes; therefore, enzymes in the CFS were further biochemically characterized. First, we measured the enzymatic activity of CFS in response to variations of temperature and pH, with the first spanning from 30 to 80 °C and the second from 3.0 to 11.0. Xylanases secreted by the microbiome CMC-50 are catalytically active from 30 °C to 75 °C, with a temperature optimum at 60 °C (Figure 1A). Considering these results, the assays to determine the pH optimum were performed at 60 °C. As shown in Figure 1B, the optimum of activity was reached at pH 7 for the CFS obtained from a culture of the microbiome CMC-50, with an almost complete loss of activity at pH < 4. Depending on the type of buffer chosen for the assay, enzymes retained around 20% of activity even in strong acidic or alkaline conditions (pH 4 or pH 11). The phenomenon where an enzyme exhibits different levels of activity or stability at the same pH but in different buffer solutions can be primarily attributed due to the buffer’s ionic strength, composition, and specific interactions with the enzyme. Enzymes in the CFS showed a preference for Tris-HCl over other buffers at the same pH value; thus, this buffer was used for further analyses. The relative thermostability over time of the CMC-50 CFS was tested at the optimum temperature (60 °C), as well as at 5 and 10 °C below it (Figure 1C). At 60 °C, enzymes retained more than 60% of their initial activity for at least 5 h. At 55 °C, the enzymes showed a consistent drop in activity around 30 h, maintaining around 40% of the initial activity up to 70 h of incubation. At 50 °C, the CFS retained more than 50% of the initial activity after 80 h. To compare the stability of these xylanases with that of a commercial enzyme mixture, we tested the thermostability of Cellic^®^ CTec2 (Novozymes A/S, Bagsvard, Denmark) at 50 °C (temperature optimum) as well as at 40 °C. A rapid decrease in activity (from 100% to 20%) after 1 h of incubation at 50 °C was observed, whereas at 40 °C, the Cellic^®^ CTec2 enzyme mixture completely lost activity within 24 h. These results demonstrated that, in the tested assay conditions, xylanases in the microbial CFS are more thermostable than those in the commercial mixture (Figure 1C). Moreover, we studied the stability over time at the storage temperature (i.e., the shelf-life at 4 °C), showing no apparent decrease in activity over a period of about 2 months (Figure 1D).

**Figure 2:**
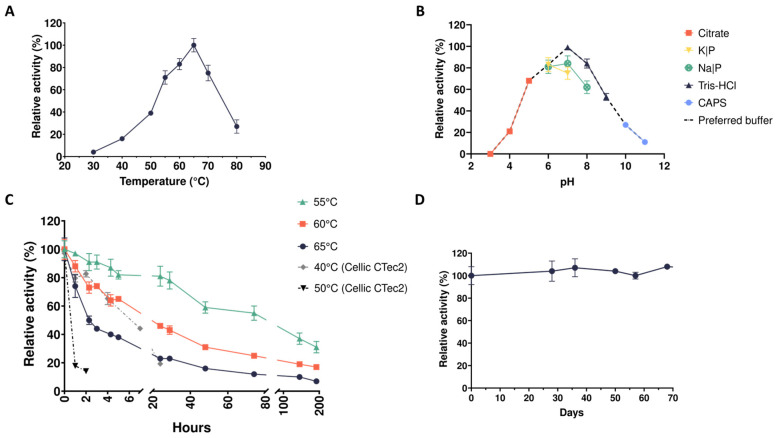
Biochemical characterization of xylanase activities in the secretome from the microbiome XYL-50. (**A**) Temperature optimum; (**B**) pH optimum; (**C**) thermostability; (**D**) storage stability.

On the other hand, xylanases secreted by the microbiome XYL-50 were active over the temperature range 40–80 °C and showed a temperature optimum at 65 °C. In this case, enzymes are slightly more thermophilic, retaining almost 80% of their maximal activity at 70 °C (Figure 2A). As for the CMC-50 CFS, enzymes in the XYL-50 CFS showed a preference for Tris-HCl pH 7; therefore, this buffer was chosen for further biochemical analyses (Figure 2B). Enzyme activity was retained above 60% of the initial one for almost all tested buffer solutions between pH 5 and 8. However, unlike xylanases in the CMC-50 CFS that retained a similar degree of activities (around 80%) at pH values of 5 and 6, enzymes in the CFS of XYL-50 showed a higher activity at pH 6 than at pH 5 (Figure 2B). The thermostability of enzymes secreted by the microbiome XYL-50 was tested at their optimum temperature (65 °C), as well as 5 and 10 °C below it (Figure 2C). At 65 °C, enzymes retained more than 50% of their initial activity for at least 2 h of incubation. At 60 °C, they showed an activity above 60% for at least 5 h, and higher than 40% for more than one day. At 55 °C, the activity was above 80% for 24 h. As in the case of CMC-50 CFS, enzymes secreted by the microbiome XYL-50 seemed to be more thermostable than those of the commercial mixture (Figure 2C). Considering the shelf-life of XYL-50 enzymes, the activity was almost unchanged over a period of two months (Figure 2D).

As already shown for the enzymes secreted by previous characterized mesophilic consortia [25], these results are promising, if we consider that the enzymes are part of a complex medium (SMS + digestate) and have not been purified. For this reason, they could perform better in terms of catalytic activity and storage stability if included in formulated enzyme mixtures. In addition, for both thermophilic microbiomes, secreted enzymes showed temperature optima close to those of enzyme cocktails that are typically used in second-generation biorefineries for LCB saccharification. Therefore, these enzymes could be also useful for improving the hydrolysis of hemicelluloses by commercial enzymatic mixtures.

### 2.2. Quantification of the Xylanolytic Activity in Cell Free Supernatants

The xylanolytic activity of microbial enzymes, measured in terms of enzyme units per milliliter of medium (U/mL), was determined via DNS assay, using pure xylan as the substrate. One xylanase unit (U) is defined as the amount of enzyme required to release one micromole (μmol) of xylose per minute of reaction (1U = 1 μmol/min). As shown in Figure S1, a calibration curve was built by carrying out the DNS assay on standard samples containing a known amount of xylose (in μmol). The DNS assay was performed at optimal conditions of temperature and pH, i.e., at 60 °C for one hour for CMC-50, and at 65 °C for 30 min for XYL-50. By measuring the absorbance at 570 nm, the micromoles (μmol) of released xylose were determined by means of the calibration curve (Figure S1). Enzyme activity values (U/mL) of the tested CFS were calculated considering both the volume of supernatant used for the assay and the incubation time (see insert Figure S1; CMC-50 = 0.33 U/mL ± 0.07 and XYL-50 = 0.62 U/mL ± 0.07).

### 2.3. Activity Profiling of Xylanases via Electrophoresis and Zymography

**Figure 3:**
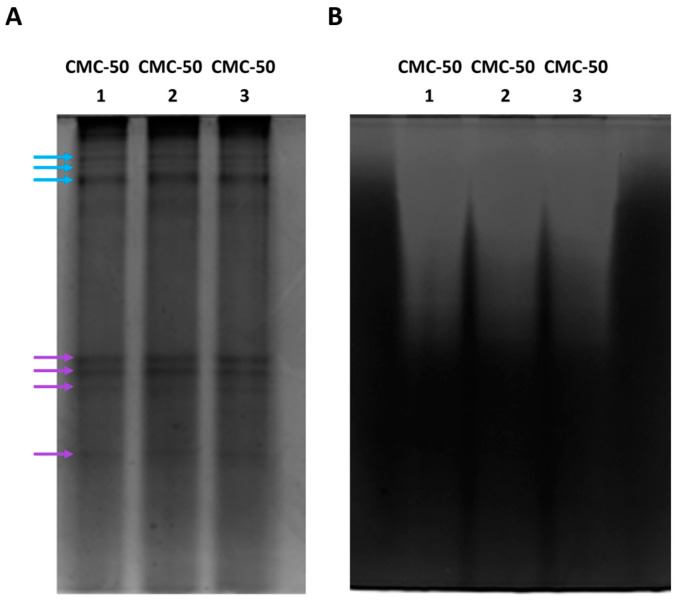
Native PAGE (**A**) and zymogram (**B**) analyses of three independently produced secretomes from the microbiome CMC-50.

Additional support to investigate the spectrum of xylanases present in the CFS was obtained by performing a native SDS-PAGE as well as an in-gel enzymatic activity visualization, i.e., zymography (Figure 3A,B). Native SDS-PAGE of CMC-50 secretomes showed the presence of protein bands at the top of the gel (Figure 3A, light-blue arrows). Zymography confirmed that some of these proteins are xylanases; in fact, an activity smear is visible in the same gel area of the corresponding zymogram (Figure 3B). Nevertheless, the presence of at least four protein bands in the middle-lower area of the native PAGE (Figure 3A, purple arrows) highlighted the presence of other proteins that do not show detectable xylanase activity in the zymogram (Figure 3B).

**Figure 4:**
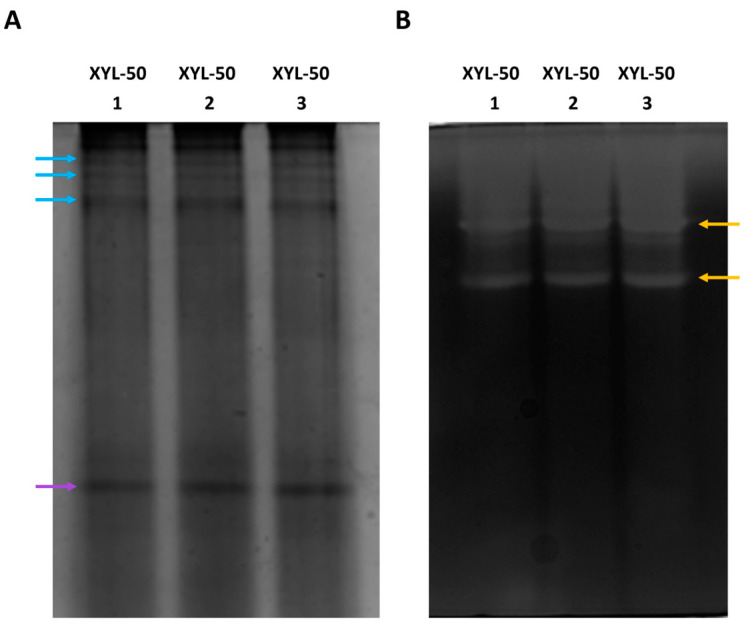
Native PAGE (**A**) and zymogram (**B**) analyses of three independently produced secretomes from the microbiome XYL-50.

Regarding the CFS of the XYL-50 microbiome, the upper area of the native PAGE revealed a band pattern similar to that of the CMC-50 secretome (Figure 4A, light-blue arrows, and only an intense protein band in the middle-lower area (Figure 4A, purple arrows). This result is consistent with the zymography, where an activity smear is visible at the top of the gel. However, two additional activity bands were detected in the zymogram (Figure 4B, yellow arrows); this could indicate that some protein bands that fall below the limit of detection of the Coomassie staining (Figure 4A) are indeed catalytically active in the zymogram (Figure 4B, yellow arrows).

### 2.4. Hydrolytic Potential of Cell Free Supernatants towards Lignocellulose-Derived Polysaccharides and Spent Mushroom Substrate

**Figure 5:**
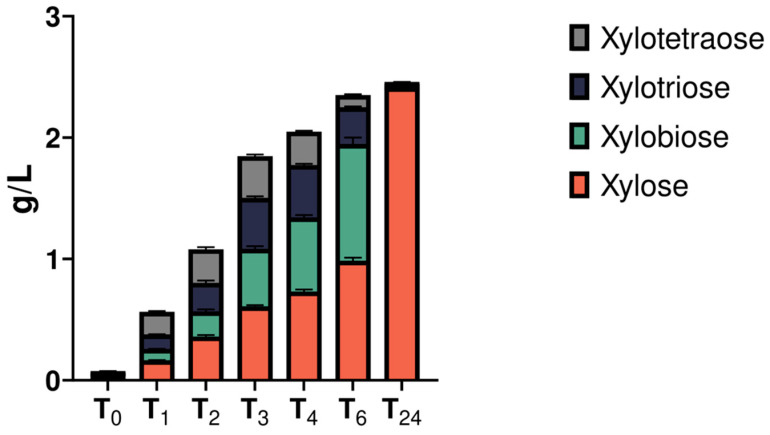
Time course of xylan degradation products obtained using the secretome of the consortium XYL-50. Each bar represents the concentration (grams/liter) of xylose (red), xylobiose (green), xylotriose (blue), and xylotetraose (grey) released after 0, 1, 2, 3, 4, 6, and 24 h of digestion.

To obtain a clearer indication of the type of secreted xylanases, a time-course digestion assay of pure beechwood xylan was performed using the XYL-50 secretome. The reactions were stopped after 1, 2, 3, 4, 6 and 24 h of digestion, and products were analyzed via high-performance anion-exchange chromatography with pulsed amperometric detection (HPAEC-PAD). As shown in Figure 5 and Figure S2, and Table S1, enzymatic xylan hydrolysis led to the production of xylose and oligosaccharides with different degrees of polymerization (DP). If compared with standards (Megazyme, Bray, Ireland), these oligoes corresponded to dimers (i.e., xylobiose), trimers (i.e., xylotriose), and tetramers (i.e., xylotetraose), with retention times of 9.8, 13.0, and 16.8, respectively (Figure S2). As depicted from the chromatogram (i.e., quantitative data analysis), at up to 2 h of incubation, the concentration of oligoes increased likely by means of the action of endo-1,4-β-xylanases. Later, the activity of the exo-1,4-β-xylosidases led to an increase in the concentration of the two oligoes with lower DP (DP2 and DP3), in parallel with a decrease in the longer DP (DP4), reaching an almost complete conversion to xylose after 24 h of incubation (Figure 5). Overall, this product profiling revealed the presence of both endo-1,4-β-xylanases and exo-1,4-β-xylosidases in the CFS (Figure 5).

**Figure 6:**
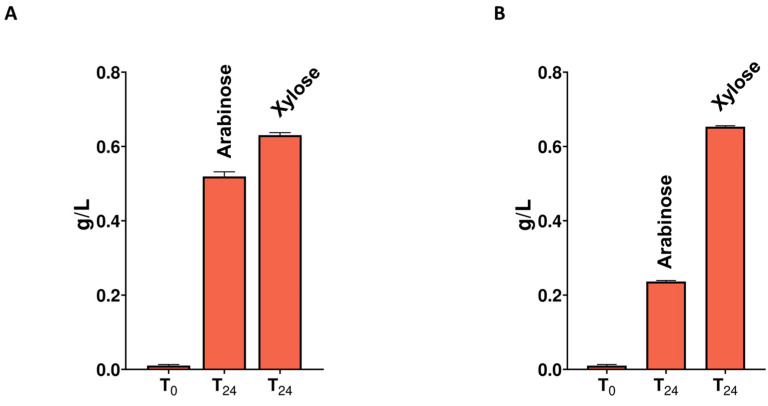
Arabinoxylan degradation profiles after digestions using the secretomes of the consortia CMC-50 (**A**) and XYL-50 (**B**). Each bar represents the concentration (g/L) of arabinose and xylose released after 0 and 24 h of digestion.

CMC-50 and XYL-50 secretomes were also tested for their hydrolytic activity on wheat flour arabinoxylan (P-WAXYL, Megazyme, Bray, Ireland), tamarind xyloglucan (P-XYGLN, Megazyme, Bray, Ireland), and ivory nut mannan (P-MANIV, Megazyme, Bray, Ireland), (Figure S3). No activity was measured towards tamarind xyloglucan, confirming the absence of soluble enzyme activity towards polysaccharides with a glucose backbone. On the other hand, both secretomes were active towards wheat flour arabinoxylan (Figure S3), releasing xylose and arabinose (Figure 6), thus indicating the presence of α-L-arabinofuranosidases in the CFS. Moreover, only the CFS of the CMC-50 microbiome showed endo-1,4 β-mannanase activity, which caused the release of oligosaccharides from ivory nut mannan (Figure S3). The hydrolytic potential of CMC-50 and XYL-50 secretomes was also evaluated on pretreated spent mushroom substrate (SMS), whose composition was already assessed in our previous work [25]. As shown in Figures S4 and S5, both secretomes were active on SMS, causing the release of oligoes with lower DP (mainly xylobiose and xylotriose) after 2 h of digestion. Later, enzyme activity led to an increase in small-molecular-weight products, with a prevalence of xylobiose and xylose after 24 h of digestion (Figures S4 and S5). These results demonstrate that CMC-50 and XYL-50 secretomes are active not only on pure substrates, but also on a complex biomass, broadening the spectrum of industrial application of these enzymes.

### 2.5. Phylogenetic and Comparative Genomic Analyses Reveal Strain-Level Differences between Microbiomes

To discern the variations in degradation performances between the XYL-50 and CMC-50 microbiomes, a comprehensive phylogenetic analysis was conducted. This computation approach aimed to unravel the intricate relationships and evolutionary distinctions within the microbial compositions of these two samples. By delving into the phylogenetic tree, we sought to gain insights into the genetic diversity and potential functional differences that might underlie the observed variations in degradation capabilities between the XYL-50 and CMC-50 microbiomes.

**Figure 7:**
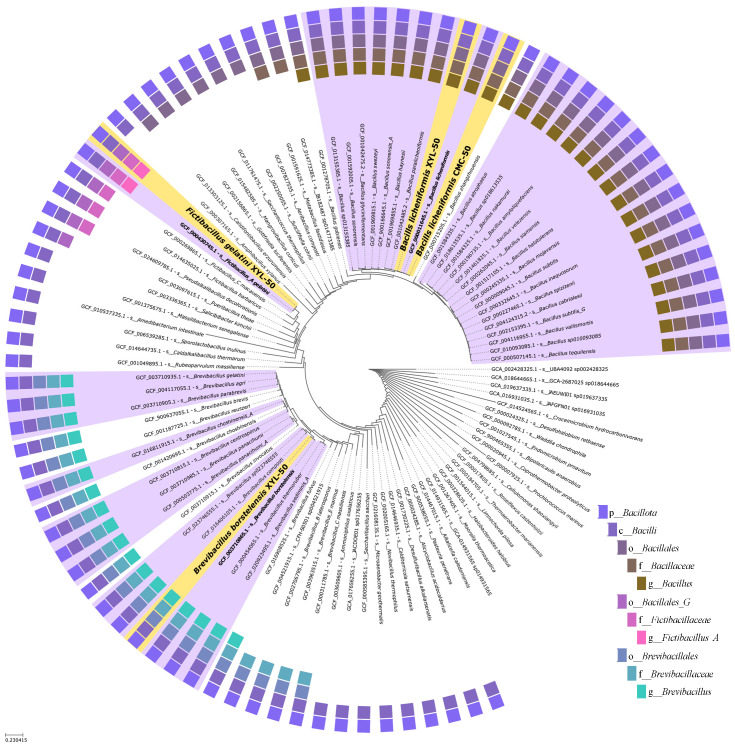
Phylogenetic tree generated using Classify Microbes with GTDB-Tk. The taxonomic membership of each leaf is represented by a color key corresponding to the outer circle on the plot. The queries are highlighted in yellow, while proximal lineages identified by GTDB-Tk are depicted in lavender.

**Figure 8:**
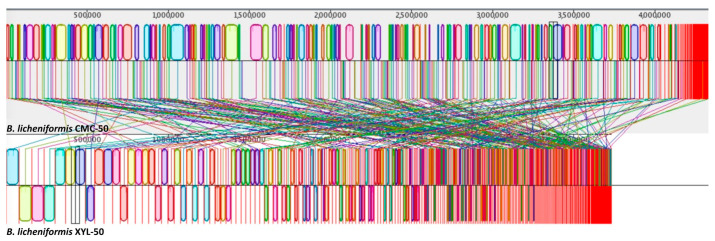
Mauve progressive alignment of *Bacillus licheniformis* CMC-50 and XYL-50.

From this analysis, it is evident that the microbiome selected on pure xylan is more heterogeneous and is composed of the following bins: *Bacillus licheniformis*, *Fictibacillus gelatini*, and *Brevibacillus borstelensis*. Surprisingly, the microbiome selected on pure CMC included only a single microorganism, *Bacillus licheniformis*, which appears to cluster together with that from XYL-50 (Figure 7). Given the close evolutionary relationship between the two microbes, we sought to determine whether *Bacillus licheniformis* CMC-50 and XYL-50 are strictly correlated. To dissect the sequence-level differences between the strains in greater detail, we utilized the bioinformatic tool Mauve. The graphical analysis, derived from nine steps of progressive alignments, reveals the presence of well-conserved regions (Figure 8). However, the two metagenome-assembled genomes (MAGs) have a distinct coverage (Figure 8) and a different distribution of contigue lengths, as also described in Bombardi et al., 2024 [25].

To assess the degree of diversity of aligned regions of the MAGs, we calculated the average nucleotide identity (ANI). Notably, the ANI value was found to be 99.8%. To corroborate this result, the Genome-to-Genome Distance Calculator (GGDC) was employed. Analysis using a generalized linear model (GLM) revealed a digital DNA-DNA hybridization (DDH) value of 99.50%, indicating that they belong to the same species. However, the value calculated to estimate the probability that the two bacteria belonged to the same sub-species was 79.29%, equal to the threshold. This is compatible with two closely related but distinct strains of *Bacillus licheniformis*.

### 2.6. Metabolic Potential and Putative Enzymatic Activities of the Thermophilic Microbiomes

**Figure 9:**
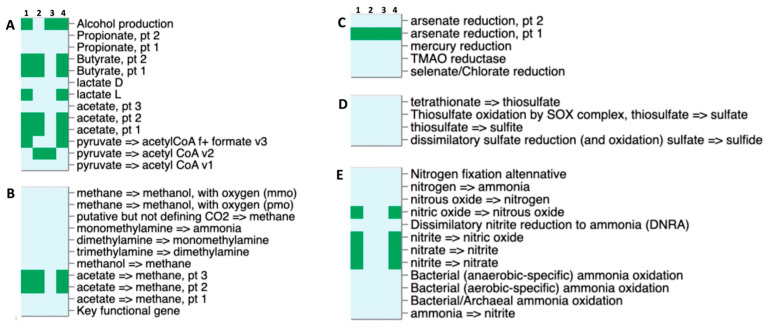
Genome comparison among bins extracted from the microbiomes CMC-50 and XYL-50. (Panel **A**) short-chain fatty acid and alcohol conversions; (Panel **B**) methanogenesis and methanotrophy; (Panel **C**) other reductases; (Panel **D**) sulfur metabolism; and (Panel **E**) nitrogen metabolism. Numbers at the top indicate (1) *Bacillus licheniformis* CMC-50; (2) *Fictibacillus gelatini* XYL-50; (3) *Brevibacillus borstelensis* XYL-50 and (4) *Bacillus licheniformis* XYL-50.

Ecosystem-relevant metabolisms across input genomes were analyzed using the DRAM tool, and the results are summarized and visualized in Figure 9. While no genes associated with sulfur metabolism were detected, a complete pathway for nitrate–nitrite–nitric oxide–nitrous oxide was identified in both *Bacillus licheniformis* CMC-50 and XYL-50. This finding suggests the potential for ammonification and denitrification processes in both the communities stabilized at 50 °C; this is consistent with the origin of these microbes that were selected from an anaerobic digestor [26,27]. All bins exhibit the potential to convert arsenate into arsenite, due to the presence of a putative arsenate reductase across all samples. However, no other metal reductase genes were identified. Additionally, except for *Brevibacillus borstelensis* XYL-50, all bins display an incomplete pathway for methane production due to the lack of the acetyl-CoA synthase. This enzyme is crucial for the reduction of carbon dioxide into acetate, a precursor for methane production. Intriguingly, the same bins (i.e., 1, 2, and 4) also harbor gene determinants for phosphate butyryltransferase (butanoyl-CoA + phosphate ⇄ CoA + butanoyl phosphate) and butyrate kinase (ADP + butyryl-phosphate ⇄ ATP + butyrate), indicating their involvement in the butyrate synthesis pathways from acetyl-CoA. Concerning fermentation pathways, *Fictibacillus gelatini* XYL-50 is unique in not showing any alcohol dehydrogenase. As expected, both *Bacillus licheniformis* XYL-50 and CMC-50 possess L-lactic dehydrogenase genes, suggesting the potential production of L-lactic acid under anaerobic conditions. Furthermore, these two bins also possess a formate lyase that produces acetyl-CoA and formate from pyruvate, with application in hydrogen storage and transport. In conclusion, the comprehensive analysis of ecosystem-relevant metabolisms using the DRAM tool provided valuable insights into the functional potential of microbial communities capable to grow on SMS at 50 °C.

**Figure 10:**
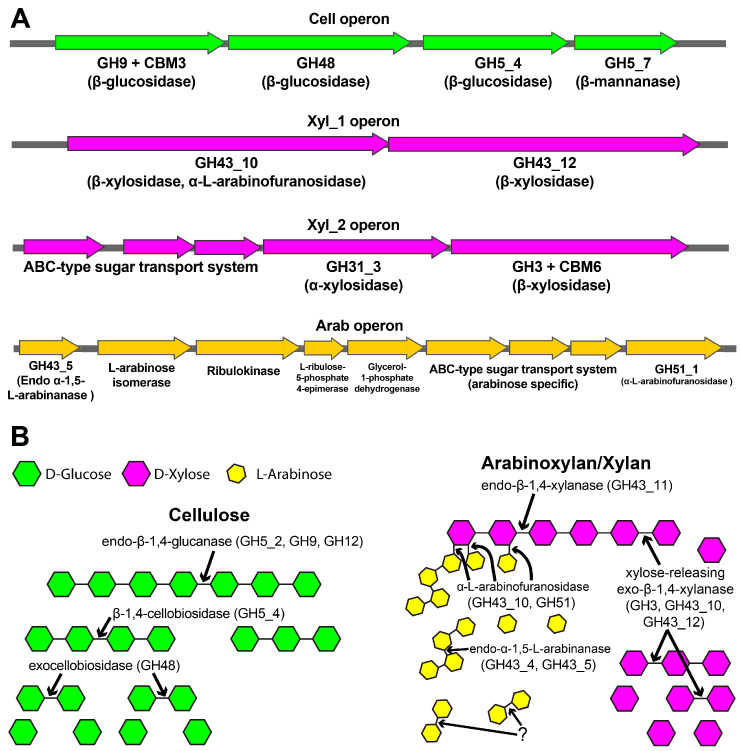
(**A**) List of operons with CAZymes involved in CMC (Cell operon), xylan/arabinoxylan (Xyl_1, Xyl_2 operons) and arabinan degradation (Arab operon) in the *Bacillus licheniformis* bins of CMC-50 and XYL-50 microbiomes. (**B**) Cellulose and xylan/arabinoxylan depolymerization routes proposed from the annotated CAZymes. Predictions of the CAZymes’ mode of action are reported in Table 1. “?” indicates that no enzyme predicted to hydrolyze such a bond was identified.

To find correlations between the measured polymers’ degradation capacities and the genetic potential of the selected microbiomes, we identified the operons and sequences coding for GHs belonging to families previously shown to possess degradation activity on CMC and xylan/arabinoxylan an arabinan (see Section 4). From this analysis, it emerged that *Brevibacillus borstelensis* and *Fictibacillus gelatini* in the XYL-50 community did not harbor genes belonging to β-glucosidase, β-xylanase/arabinoxylanase, or α-L-arabinanase, but only GH3 genes predicted to be N-acetyl-glucosaminidase. Therefore, these strains may be commensal species that can grow on the products of xylan depolymerization by enzymes produced by other microbiome members. On contrary, metagenomic assemblies of *Bacillus licheniformis* strains from both microbiomes contain four different operons (Figure 10A) and eleven CAZymes (Table 1) with a predicted functional diversity sufficient to complete the depolymerization of both xylan and CMC (Figure 10B).

Nonetheless, there are some differences: the GH43_10 sequence (Xyl_1 operon, Figure 10A) was found only in *Bacillus licheniformis* CMC-50 but was absent from the corresponding operon of *Bacillus licheniformis* XYL-50, probably due to superior sequence fragmentation of the latter assembly. Moreover, besides the shared arabinan-degradation operon (Arab, Figure 10A), CMC-50 has other two additional GH43 coding for endo-α-1,5-L-arabinase. It is noteworthy that most of the identified sequences are very similar to previously characterized CAZymes (Table 1), making it easy to identify the routes by which the different enzyme families depolymerize the sugars (Figure 10B): CMC is depolymerized by GH5_2 (+CBM3), GH5_4, GH9 (+CBM3), and GH12, xylan/arabinoxylan by GH43_10, GH43_11, and GH51_1. The resulting low-molecular-weight oligomers of glucose and xylose are hydrolyzed to monomers by GH48 and by GH3 (+CBM6) or GH43_12. Despite the existence of arabinanase (GH43_4, GH43_5) acting on arabinan, these enzyme families are usually associated with an endo mode of action, and it is not clear how L-arabinose is finally released.

**Figure 11:**
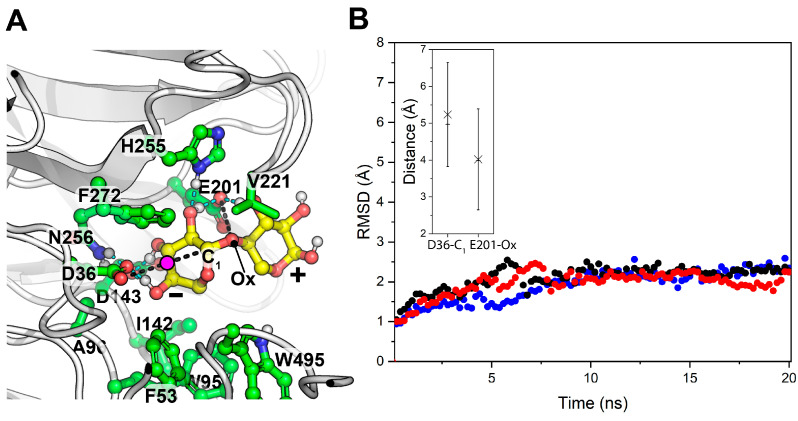
Simulated xylobiose interaction with GH43_10. MD simulation frame with the higher binding affinity after MD refinement of the GH43_10 AF model docked with xylobiose (**A**): the model is visualized as a ball-and-stick sketch with interacting residues and xylobiose; carbon atoms of the protein model and xylobiose are differently colored, while oxygen, nitrogen, and polar hydrogens are shown in red, blue, and white; hydrogen bonds are marked as cyan dashed lines; non-covalent interactions of catalytic residues to reactive xylobiose atoms are marked as black dashed lines; a magenta circle is used to indicate the putative position of the water molecule required to be activated for the nucleophilic attack to C_1_, as shown in [40]. MD simulation statistics (**B**): different replicates are depicted with different colors. Only frames from 10 to 20 ns were used to generate the distance plots of the atoms involved in the catalysis (marked as black dashed lines in panel (**A**)); the average and the median values are depicted with a cross and a horizontal line, respectively. C_1_: carbon 1 atom of reducing-end sugar moiety; Ox: oxygen atom.

GH43_10 is the only protein for which the sequence-based functional annotation as xylanase is not consistent between the predictors used. To obtain a more reliable function prediction, a structure-based approach was used combining AlphaFold, a deep learning model, to dynamically dock a model xylobiose in the active site, and a molecular dynamics (MD) refinement to confirm the physics compliance and stability of the prediction between the enzyme model and the substrate. The modeling results indicate the formation of a stable GH43_10-xylobiose complex in a catalytically competent binding mode (Figure 11). Moreover, the binding mode with the higher affinity from the sampled conformers suggests that the oxygen of the catalytic D36 does not directly make a nucleophilic attack on the distant C_1_ of the reducing end sugar moiety, but activates a water molecule in-between. This model is in high agreement with the most common transition state predicted for GH43 xylanase, as obtained from a recent quantum mechanics/molecular mechanics simulation approach [40]. Considering that GH43_10 shares ≈50% identity with a characterized bifunctional β-xylosidase/α-L-arabinofuranosidase from the thermophile *Caldicellulosiruptor saccharolyticus* DSM8903 [34], and belongs to a xylan-degrading operon with another exo-β-xylanase (Figure 10), it is suggested that, in the XYL-50 microbiome, GH43_10 is important for complete and synergistic depolymerization of xylan and arabinoxylan oligomers, after the extracellular action of the endo-β-xylanase GH43_11 (Table 1).

## 3. Discussion

The flourishing agro-industrial activities over the last few decades have not only significantly enhanced our capacity to produce food and other resources, but have also led to an exponential increase in the production of lignocellulose biomasses (LCB). As abundant and cost-effective feedstocks, LCB primarily serve as sources for fermentable sugars, chemicals, and various renewable products. The traditional approaches for LCB hydrolysis in industrial settings, involving harsh methodologies like acid hydrolysis or thermal shock, are not only costly due to the high solvent usage, but also generate toxic compounds that inhibit microbial fermentation processes. In response, the scientific community has started exploring the use of microbial consortia for LCB deconstruction. This method leverages the natural abilities of microorganisms to efficiently transform LCB into organic products, marking a significant shift towards more sustainable practices. In this regard, thermophilic microorganisms have attracted significant interest. Their ability to produce thermostable enzymes capable of withstanding harsh industrial conditions, without compromising their activity or stability, presents a promising avenue for lignocellulose conversion.

The results of this study highlight the significant potential of microbial consortia (i.e., microbiomes) isolated from relevant industrial environments to produce robust enzymes, with promising implications for industrial applications, such as in biorefineries. The microbiomes, selected at 50 °C using carboxymethyl cellulose (CMC-50) and xylan (XYL-50), exhibited the secretion of xylanolytic enzymes that demonstrated a broad range of temperature and pH activity. Notably, the optimal activity conditions for these xylanases were well aligned with the operational conditions of current enzyme cocktails used in biorefineries, thereby presenting an opportunity to integrate these enzymes into existing bioprocesses. The thermostability profile of xylanases from CMC-50 and XYL-50 is particularly interesting, with enzymes retaining substantial activity even after prolonged exposure to temperatures close to their respective optima. This is a crucial feature for industrial enzymes, as it suggests that they could be effective over extended periods of time, reducing overall production costs. In comparison to a commercial enzyme mixture, Cellic^®^ CTec2, xylanases from selected microbiomes showed superior stability, in the same experimental conditions, which is critical for processes requiring high thermal endurance.

The assays conducted to determine enzymatic activity, as well as the electrophoretic and zymographic analyses, provided further insights into the enzyme profiles and activities within the secretomes. The presence of multiple xylanases, as indicated by the band patterns and activity smears in zymograms, suggests the presence of different enzymes that could be harnessed to enhance the breakdown of complex polysaccharides. This is complemented by the hydrolytic potential demonstrated towards other LCB-derived polysaccharides and spent mushroom substrate (SMS), indicating that these enzymes can effectively convert a range of biomass substrates (such as flour arabinoxylan and ivory nut mannan) into either oligosaccharides or simpler fermentable sugars. This feature, combined with their thermostability, positions these enzymes as valuable tools for the deconstruction of plant biomass in sustainable biofuel and biochemical production. Furthermore, the robustness of these enzymes, reflected in their stability under various conditions and their ability to operate effectively within a complex medium without the need for purification, suggests they could be cost-effectively produced and utilized in industrial settings. This could potentially lead to more sustainable and economically viable processes in the biorefinery sector.

On the other hand, the phylogenetic and comparative genomic analysis conducted in this study sheds light on the genetic diversity and functional potential underlying the observed variations in degradation capabilities between the XYL-50 and CMC-50 microbiomes. The analysis revealed that while the microbiome selected on pure xylan (XYL-50) exhibited greater heterogeneity, comprising *Bacillus licheniformis*, *Fictibacillus gelatini*, and *Brevibacillus borstelensis*, the microbiome selected on pure CMC (CMC-50) included only *Bacillus licheniformis*. Interestingly, despite this difference, *Bacillus licheniformis* from both communities clustered closely together in the phylogenetic tree, indicating a close evolutionary relationship. Further analysis using Mauve revealed well-conserved regions between the metagenome-assembled genomes (MAGs) of *Bacillus licheniformis* CMC-50 and XYL-50, although with distinct coverage and contig lengths. The high average nucleotide identity (ANI) of 99.8% and digital DNA-DNA hybridization (DDH) value of 99.50% suggest that these strains belong to the same species. However, the probability that they belong to the same sub-species was calculated at 79.29%, indicating distinct strains of *Bacillus licheniformis*. Functional profiling and CAZome analysis provided insights into the metabolic potential of these microbiomes. The presence of genes associated with nitrate–nitrite–nitric oxide–nitrous oxide pathways suggest the potential for ammonification and denitrification processes in both communities. Additionally, the detection of genes involved in arsenate reduction and phosphate butyryltransferase/butyrate kinase pathways highlights their metabolic versatility. Notably, the absence of genes for methane production in some bins suggests distinct metabolic pathways among community members.

The identification of specific glycoside hydrolases (GH) associated with CMC and xylan degradation provides a link between genetic potential and polymer degradation capacities. *Bacillus licheniformis* strains from both communities were found to possess operons and CAZymes necessary for complete depolymerization of both CMC and xylan, suggesting their pivotal role in biomass degradation. The structure-based approach used to predict the function of GH43_10 as a xylanase in the XYL-50 microbiome further supports the synergistic depolymerization of xylan and arabinoxylan oligomers. Overall, the integration of phylogenetic, genomic, and functional analyses enriches our understanding of the microbial consortia and their enzymatic capabilities. These insights provide a foundation for future studies aiming to optimize enzyme production and harness the metabolic potential of thermophilic microbial communities for biotechnological applications, particularly in biomass conversion processes.

In conclusion, the findings from this study underline the potential of using these thermophilic microbiomes as a source of efficient, stable, and broad-spectrum xylanases for industrial applications. Future studies should focus on scaling up the production and further characterizing the enzyme mixtures, as well as testing their efficacy in industrial-scale biomass conversion processes. This could pave the way for the development of more efficient and cost-effective bioconversion processes, ultimately contributing to the advancement of sustainable biotechnologies.

## 4. Materials and Methods

### 4.1. Media and Chemicals

Spent mushroom substrate (SMS) and the digestate from an anaerobic digestor were obtained from the agro-zootechnical farm “La Torre” (Isola della Scala, Verona, Italy) and pretreated as reported by Bombardi et al., 2024 [25]. Briefly, SMS was oven-dried at 105 °C for about 16 h, ground to increase the available surface area, and passed through a 2 mm sieve (Giuliani Tecnologie, Torino, Italy) to select for more uniform particles size. The digestate was centrifuged at 6000× *g* for ten minutes to remove the bulk of solid particles. Both were sterilized by autoclavation at 121 °C for 60 min. Microbial consortia (i.e., microbiomes) previously enriched on carboxymethylcellulose (CMC) or corncob xylan (XYL), i.e., CMC-50 and XYL-50, were revitalized from glycerol stocks and proliferated at 50 °C in seed media containing 0.5% *w*/*v* of yeast extract (VWR Chemicals, Milano, Italy) and either 0.5% *w*/*v* of CMC (VWR Chemicals, Milano, Italy) or 0.5% *w*/*v* of XYL (TCI Chemicals, Shanghai, China), respectively.

### 4.2. Microbial Growth for Enzyme Production

To induce enzyme secretion, microbiomes derived from the seed cultures were reinoculated in triplicate in an induction medium prepared by mixing sterile digestate and SMS as the sole carbon source, and incubated at 50 °C. Cultures were sampled daily, centrifuged at 6000× *g* for 5 min to obtain cell-free supernatants (CFS), and subjected to colorimetric Azo-xylan assay (Megazyme, Bray, Ireland) to measure the xylanase activity (see Section 4.3). After a few days of cultivation, 10 mL of the culture that had reached the highest activity was used to inoculate three flasks containing 100 mL of freshly prepared induction medium prepared by mixing 5% (*w*/*v*) of sterile SMS in sterile and tenfold-diluted digestate, which was previously centrifuged at 6000× *g* for ten minutes to remove solid particles. As measured in Bombardi et al., 2024 [25], the SMS composition is 34.24% (*w*/*w*) cellulose, 19.45% (*w*/*w*) hemicellulose, 17.73% (*w*/*w*) lignin, and 13.81% (*w*/*w*) ashes, whereas the digestate has a total solid (TS) content of 7.04% (*w*/*w*), a total volatile solid (TVS) content of 4.11% (*w*/*w*), a total carbon (TC) content of 29.55% (*w*/*w*), a total nitrogen (TN) content of 3.00% (*w*/*w*), a total sulfur (TS) content of 1.13% (*w*/*w*), a total hydrogen (TH) content of 4.11% (*w*/*w*), and a carbon/nitrogen (C/N) ratio of 9.85. Maximum enzymatic activity was generally achieved after two or three days of cultivation. Therefore, cultures were centrifuged twice at 6000× *g* for 10 min, to remove the bulk of the remaining SMS, and filtered through a 0.22 μm nylon membrane by means of a vacuum-filtration apparatus (LLG-Labware, Meckenheim, Germany). The obtained CFS were stored at 4 °C until further characterizations.

### 4.3. Azo-Xylan Assay

CFS (i.e., the secretomes) of CMC-50 and XYL-50 microbiomes were characterized in terms of temperature and pH optima, as well as for their thermostability and storage stability, by monitoring their endo-1,4-β-D-xylanase activity using Azo-Xylan (Megazyme, Bray, Ireland) as the substrate. Enzyme assays were carried out following the manufacturer’s instructions (Megazyme, Bray, Ireland), with few modifications. Briefly, 50 μL aliquots of 1% *w*/*v* Azo-Xylan and 50 μL of CFS were preincubated for 5 min at 50 °C. The reactions were started by mixing prewarmed Azo substrate and prewarmed CFS and incubating for two hours at 50 °C. Then, reactions were stopped by adding 250 μL of precipitating solution (95% *v*/*v* ethanol) and left for 10 min at room temperature. In parallel, negative controls were prepared by directly mixing the precipitating solution and the Azo-Xylan solutions before adding the CFS. Then, samples were centrifuged for 5 min at 10,000× *g* to remove the non-hydrolyzed polymeric substrate, and 200 μL of clarified reaction mixture was transferred into a well of a 96-well microplate (Sarstedt, Numbrecht, Germany). The xylanase activity was determined by measuring the absorbance at 590 nm using a Synergy Neo2 Hybrid Multi-Mode Reader (Agilent Technologies, Santa Clara, CA, USA) with reference to a standard curve (Cellic Ctec2 Novozymes, Bagsvard, Denmark). All experiments were run in triplicate. To study the enzyme thermostability, CFS were incubated at their optimal temperature as well as at 5 °C or 10 °C below. Aliquots were withdrawn at different time points throughout the incubation to carry out Azo-Xylan assays at their optimal temperature and pH of activity. The decrease in enzyme activity over time of the CFS was expressed as the percentage of retained activity by setting as 100% that of the same CFS tested without any prelaminar incubation (t = 0).

### 4.4. Reducing Sugars Assay for Enzyme Units Measurement

The concentration of reducing sugars was estimated by 3,5-dinitrosalicylic (DNS) assay [41]. In brief, the assay was performed by mixing the same volumes (35 µL) of 1% (*w*/*v*) xylan (P-XYLNBE, Megazyme, Bray, Ireland) solution and microbial CFS. Two control reactions (enzyme and substrate control) were set up to measure the inherent concentration of reducing sugars in the CFS and in the xylan solution. After stopping the reaction by adding DNS solution, the absorbance at 570 nm of the samples was measured and used to determine the micromoles of released xylose by means of a calibration curve. The enzyme activity (U/mL) was calculated by applying the following equation:

$$\frac{U}{\mathrm{mL}}=\frac{\left[\mathrm{released}\;\mathrm{xylose}\;(\mu \mathrm{mol})\right]}{\left[\mathrm{reaction}\;\mathrm{time}\;\left(\min\right)\right]\times \left[\mathrm{enzyme}\;\mathrm{volume}\;\left(\mathrm{mL}\right)\right]}$$

### 4.5. Electrophoretic and Zymographic Analysis

Native polyacrylamide gel electrophoresis (native PAGE) and zymography were performed as reported in [42] with few modifications. A non-reducing loading buffer was added to the samples, and no boiling denaturation was performed before protein electrophoresis separation. For zymography, beechwood xylan 1% (*w*/*v*) (P-XYLNBE, Megazyme, Bray, Ireland) was added during the preparation of the resolving gel. Following electrophoresis, the gel was washed with a 2.5% (*v*/*v*) Triton X-100 solution and incubated at 4 °C in the desired enzyme assay buffer for 10 min. Then, the enzyme reaction was started by increasing the temperature to the enzyme’s optimum temperature and incubating for 7 min. The gel was then stained using a 0.1% (*w*/*v*) Congo Red solution for 30 min at room temperature. After destaining by washing with 1 M NaCl for 15 min, areas of xylan hydrolysis appeared as clear bands against a darkly stained background.

### 4.6. Hydrolysis of LCB-Derived Polysaccharides

The enzymatic activity of CMC-50 and XYL-50 secretomes was tested towards Beechwood xylan (P-XYLNBE, Megazyme, Bray, Ireland), wheat flour arabinoxylan (P-WAXYL, Megazyme, Bray, Ireland), tamarind xyloglucan (P-XYGLN, Megazyme, Bray, Ireland), ivory nut Mannan (P-MANIV, Megazyme, Bray, Ireland) and spent mushroom substrate (SMS). SMS was pretreated in 1% NaOH, autoclaved, and milled before use. Samples were prepared by directly mixing 1 mL of CFS containing microbial enzymes and the substrates at the final concentration of 1% (*w*/*v*). For SMS digestions, 900 µL of CFS was mixed with 100 µL of 0.5 M Tris-HCl pH 7.0 and the mix added to the substrate at a final concentration of 10% (*w*/*v*). Digestions were performed at the optimal temperature and pH for each secretome under constant shaking of 800 rpm in an LLG-uniTHERMIX2 pro thermos shaker (Lab Logistics Group GmbH, Meckenheim, Germany). Hydrolysis was stopped after different times by inactivating the secretomes at 100 °C for 5 min. After enzyme inactivation, all samples were centrifuged at 10,000× *g* for 5 min, and the hydrolysates were stored at −20 °C for subsequent analysis.

### 4.7. HPAEC-PAD Analysis

High-performance anion-exchange chromatography with pulsed amperometric detection (HPAEC-PAD) was carried out on a Dionex ICS-6000 system (Thermo Fisher Scientific, Waltham, MA, USA) equipped with a Palladium Hydrogen (PdH) reference electrode, and a gold working electrode for the detection [43]. HPAEC-PAD experiments on pure substrates were performed on a CarboPac PA210-Fast-4 μm column (Thermo Fisher Scientific, Waltham, MA, USA) using a gradient of potassium hydroxide from 12 mM to 100 mM, with a flow rate of 0.6 mL/min, and the temperature of the auto-sampler and the column set at 10 and 30 °C, respectively. SMS digestions were analyzed on a CarboPac PA200-3 mm column, with a flow rate of 0.3 mL/min and the same temperatures for the auto-sampler and column. Initial conditions were set to 0.1 M NaOH (eluent A) followed by a linear gradient towards an increasing proportion of a solution of 0.1 M NaOH + 0.5 M NaOAc (eluent B). The gradient reached 60% of solution B in 20 min. All samples were centrifuged, diluted with dH_2_O, and filtered before use. Products of digestion were identified and quantified according to standard curves prepared in the range of 25–0.1 mg/L. All measurements were run in triplicate and the mean values ± SD are reported.

### 4.8. Phylogenetic Analysis and Functional Annotation of CAZymes

The phylogenetic tree was generated using Classify Microbes with GTDB-Tk—v2.3.2 in KBase, by using the metagenome assembly obtained using MegaHit (v1.1.3) implemented with the MetaWRAP-Assembly module [44] as described in Bombardi et al., 2024 [25]. Two bins, i.e., *Bacillus licheniformis* CMC-50 and XYL-50, were selected based on their genetic relatedness, and the differences at the sequence level were evaluated with Mauve tool (v. 2.4.0) [45]. A pairwise comparative analysis was carried out by calculating the average nucleotide identity (ANI) using FastANI (v. 1.34) [46]; the percentage threshold for species boundary is 95% ANI. In silico DNA–DNA hybridization (DDH) values were calculated using the Genome-to-Genome Distance Calculator (GGDC) v 3.0 [47]. Carbohydrate active enzymes (CAZymes) searches and specific glycoside hydrolases (GH) family assignment were performed using the dbCAN3 metaserver [48]. Operons were predicted using the operon-mapper web server [49]. Sequences from GH families that include endo-β-glucosidases/xylanase (5, 6, 7, 8, 9, 10, 11, 12, 26, 30, 43, 45, 48, 51), and exo-β-glucosidases/xylanase (1, 3, 7, 9, 48) were further assigned to an enzymatic function with a sequence-based approach: the result of the best BLASTp hit with minimum 40% identity to the database of characterized CAZymes (http://www.cazy.org/ (accessed on 25 March 2024) was combined with the deep learning CLEAN predictor of E.C. number [38]. The prediction was considered reliable if consistent between methods. Otherwise, the structure-based approach described in the next paragraph was applied.

### 4.9. Modeling of the Enzyme-Substrate Complex

The 3D molecular model of GH43_10 was predicted using AlphaFold v.2.3.2 [50], v3 model, and ColabFold v.1.5.5 (https://github.com/sokrypton/ColabFold (accessed on 5 April 2024) [51] in monomeric oligomeric state. The default ColabFold parameters were retained, allowing the use of structural templates from Protein Data Bank and a more thorough sampling by activating the dropout option and using 3 different seeds. Only monomeric models with plDDT ≥ 0.90 were retained and the best was selected. The resulting model was energy minimized by 2000 steepest descent steps within ColabFold and used for docking with xylobiose (O[C@@H]1CO[C@@H](O[C@@H]2COC(O)[C@H](O)[C@H]2O)[C@H](O)[C@H]1O) by the end-to-end deep generative method DynamicBind v.1.0 [52] to allow for full flexibility in the protein and ligand during the docking procedure, and to obtain an induced-fit complex. The top-ranked modeled complex was retained. This was chosen only between poses, with the substrate docked in a catalytically competent binding mode when distances between the carbonylic oxygen of the two catalytic residues (nucleophile and acid/base) are <4.5 Å to the C_1_ of the non-reducing-end sugar moiety and the glycosidic bond oxygen. The complex was energy minimized and refined by running three independent constant-pressure and temperature classical molecular dynamics simulations (20 ns each, saving every 0.2 ns) in TIP3P water molecules with 10 Å padding. This step was performed using OpenMM 7.7.0 [53] under the amber ff14SB force field [54], with a time step of 2.0 fs and at 50 °C. Simulation frames were analyzed with mdtraj 1.9.4 [55]. The calculation of binding free energy for the interaction in the simulated enzyme–substrate model was estimated by applying molecular mechanics energies combined with the generalized Born and surface area continuum solvation, as conducted in Orlando et al., 2021 [56].

## Figures and Tables

**Table 1:** List of metagenomic GH sequences that belong to families for which β-glucosidase, β-xylosidase, or L-α-arabinofuranosidase activity is known from the CAZy database (http://www.cazy.org/, accessed on 25 March 2024). Functional predictions are reported. E. C.: Enzyme Commission; 3.2.1.37: oligoxyloglucan hydrolase; 3.2.1.4: β-1,4-glucanase, both eso and endo; 3.2.1.151: xyloglucan-specific endo-beta-1,4-glucanase; 3.2.1.55: alpha-L-arabinofuranosidase; 3.2.1.8: Endo-β-xylosidase; 3.2.1.176: exocellobiohydrolase; 3.2.1.99: arabinan endo-1,5-alpha-L-arabinosidase. MSCH: most similar characterized homologue, defined as the top-1 Blast hit retrieved from the characterized portion of the CAZy database, using each metagenomic sequence as query. The “Operon” column refers to the naming presented in Figure 10.

Source	Family	Operon	Predicted E.C. Number ^a^	Predicted Signal Peptide ^b^	Activity MSCH (Experimental)	Uniprot ^c^ ID MSCH	Identity (%) to MSCH	Bibliography MSCH
XYL-50_1	GH3 + CBM6	Xyl_2	3.2.1.37	-	Isoprimeverose-producing Oligoxyloglucan Hydrolase	I0IUK4	45.7	[28]
CMC-50_1	GH3 + CBM6	Xyl_2	3.2.1.37	-	Isoprimeverose-producing Oligoxyloglucan Hydrolase	I0IUK4	45.7	[28]
XYL-50_1	GH5_2 + CBM3	-	3.2.1.4	Sec/SPI	Endo β-1,4-glucanase	Q7X3S6	100	[29]
CMC-50_1	GH5_2 + CBM3	-	3.2.1.4	Sec/SPI	Endo β-1,4-glucanase	Q7X3S6	100	[29]
XYL-50_1	GH5_4	Cell	3.2.1.4	Sec/SPI	β-1,4-cellobiosidase (reducing end)	AAU40777	100	[30]
CMC-50_1	GH5_4	Cell	3.2.1.4	Sec/SPI	β-1,4-cellobiosidase (reducing end)	AAU40777	100	[30]
XYL-50_1	GH9 + CBM3	Cell	3.2.1.4	Sec/SPI	Endo β-1,4-glucanase	Q6SYB5	100	[31]
CMC-50_1	GH9 + CBM3	Cell	3.2.1.4	Sec/SPI	Endo β-1,4-glucanase	Q6SYB5	100	[31]
XYL-50_1	GH12	-	3.2.1.151	Sec/SPI	Endo β-1,4-glucanase	Q7X4S4	100	[31]
CMC-50_1	GH12	-	3.2.1.151	Sec/SPI	Endo β-1,4-glucanase	Q7X4S4	100	[31]
CMC-50_1	GH43_4	-	3.2.1.99	Sec/SPII	Endo α-1,5-L-arabinanase	A0A6M4JQW9	75.5	[32]
CMC-50_1	GH43_5	Arab	3.2.1.99	Sec/SPI	Endo α-1,5-L-arabinanase	UPI000043D9A2	100	[33]
XYL-50_1	GH43_5	Arab	3.2.1.99	Sec/SPI	Endo α-1,5-L-arabinanase	UPI000043D9A2	100	[33]
CMC-50_1	GH43_5	-	3.2.1.99	Sec/SPII	Endo α-1,5-L-arabinanase	UPI000043FF33	99.1	[33]
CMC-50_1	GH43_10	Xyl_1	3.2.1.55	-	Bifunctional β-xylosidase/α-L-arabinofuranosidase	A4XGG5	50.6	[34]
XYL-50_1	GH43_11	-	3.2.1.8	Sec/SPII	Endo-β-xylosidase	D3R467	100	[35]
CMC-50_1	GH43_11	-	3.2.1.8	Sec/SPII	Endo-β-xylosidase	D3R467	100	[35]
XYL-50_1	GH43_12	Xyl_1	3.2.1.37	-	Xylose-releasing exo-β-1,4-xylanase	Q65MB6	99.1	[36]
CMC-50_1	GH43_12	Xyl_1	3.2.1.37	-	Xylose-releasing exo-β-1,4-xylanase	Q65MB6	99.1	[36]
XYL-50_1	GH48	Cell	3.2.1.176	-	exocellobiohydrolase	Q19VP0	95	[37]
CMC-50_1	GH48	Cell	3.2.1.176	-	exocellobiohydrolase	Q19VP0	95	[37]
XYL-50_1	GH51	Arab	3.2.1.55	-	α-L-arabinofuranosidase	A0A6M4JKK3	72.4	[32]
CMC-50_1	GH51	Arab	3.2.1.55	-	α-L-arabinofuranosidase	A0A6M4JKK3	72.4	[32]

^a^ Predicted with CLEAN [38]. ^b^ Predicted with SignalP 6.0 [39]. ^c^ The Uniparc ID was used in case the Uniprot ID was not available.

## Data Availability

Sequencing data have been submitted to SRA database, project PRJNA1047304.

## Supplementary Materials

The following supporting information can be downloaded at: https://www.mdpi.com/article/10.3390/ijms25189887/s1.
