# Supplementary material for: Thermophilic Hemicellulases Secreted by Microbial Consortia Selected from an Anaerobic Digester
Source: Int J Mol Sci. 2024 Sep 13;25(18):9887. doi: 10.3390/ijms25189887 (PMC11432564; doi:10.3390/ijms25189887)
Supplement: Supplementary file 1 [file ijms-25-09887-s001.zip › Table S1.pdf]

Table S1. HPAEC-PAD quantification of xylan and arabinoxylan hydrolysis products obtained using the secretome of the consortium CMC-50 and XYL-50.

Sample Name	Arabinose g/L ± S.D.	Xylose g/L ± S.D.	Xilobiose g/L ± S.D.	Xilotriose g/L ± S.D.	Xilotetraose g/L ± S.D.
**CMC-50 Xylan (T0)**	n.d.	n.d.	n.d.	0.04 ± 0.1	0.06 ± 0.02
**CMC-50 Xylan (T1)**	n.d.	0.17 ± 0.01	0.12 ± 0.01	0.17 ± 0.01	0.19 ± 0.01
**CMC-50 Xylan (T2)**	n.d.	0.33 ± 0.02	0.25 ± 0.01	0.30 ± 0.03	0.25 ± 0.04
**CMC-50 Xylan (T3)**	n.d.	0.48 ± 0.02	0.47 ± 0.05	0.39 ± 0.04	0.24 ± 0.03
**CMC-50 Xylan (T4)**	n.d.	0.80 ± 0.03	1.04 ± 0.08	0.26 ± 0.02	n.d.
**CMC-50 Xylan (T6)**	n.d.	0.79 ± 0.09	0.97 ± 0.14	0.33 ± 0.03	0.05 ± 0.01
**CMC-50 Xylan (T24)**	n.d.	2.18 ± 0.04	0.25 ± 0.04	n.d.	n.d.
**XYL-50 Xylan (T0)**	n.d.	n.d.	n.d.	0.03 ± 0.01	n.d.
**XYL-50 Xylan (T1)**	n.d.	0.16 ± 0.01	0.09 ± 0.01	0.12 ± 0.01	0.19 ± 0.01
**XYL-50 Xylan (T2)**	n.d.	0.36 ± 0.01	0.21 ± 0.02	0.24 ± 0.02	0.28 ± 0.02
**XYL-50 Xylan (T3)**	n.d.	0.61 ± 0.01	0.47 ± 0.02	0.42 ± 0.01	0.34 ± 0.01
**XYL-50 Xylan (T4)**	n.d.	0.73 ± 0.02	0.61 ± 0.02	0.43 ± 0.01	0.28 ± 0.01
**XYL-50 Xylan (T6)**	n.d.	0.98 ± 0.03	0.96 ± 0.05	0.30 ± 0.01	0.10 ± 0.01
**XYL-50 Xylan (T24)**	n.d.	2.40 ± 0.03	n.d.	n.d.	n.d.
**CMC-50 AX (T0)**	n.d.	n.d.	n.d.	n.d.	n.d.
**CMC-50 AX (T24)**	0.52 ± 0.01	0.63 ± 0.01	n.d.	n.d.	n.d.
**XYL-50 AX (T0)**	n.d.	n.d.	n.d.	n.d.	n.d.
**XYL-50 AX (T24)**	0.24 ± 0.01	0.65 ± 0.01	n.d.	n.d.	n.d.

n.d.: not detectable (i.e., below the limit of quantification – LOQ –)
S.D.: Standard deviations (*n* = 3)
