# Supplementary figures and images for: Thermophilic Hemicellulases Secreted by Microbial Consortia Selected from an Anaerobic Digester
Source: Int J Mol Sci. 2024 Sep 13;25(18):9887. doi: 10.3390/ijms25189887 (PMC11432564; doi:10.3390/ijms25189887)

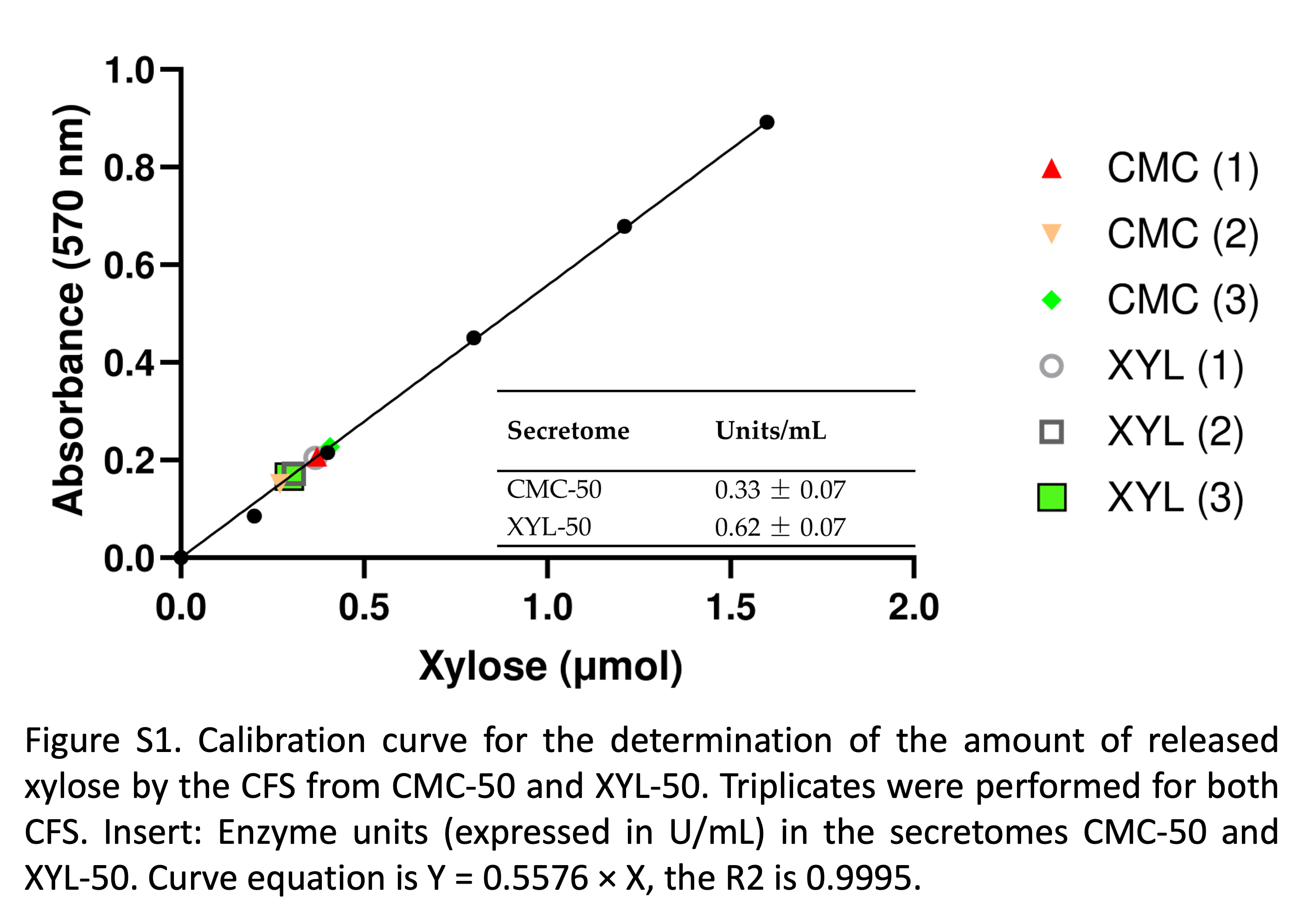

Supplement: Supplementary file 1 [file ijms-25-09887-s001.zip › Figure S1.png]

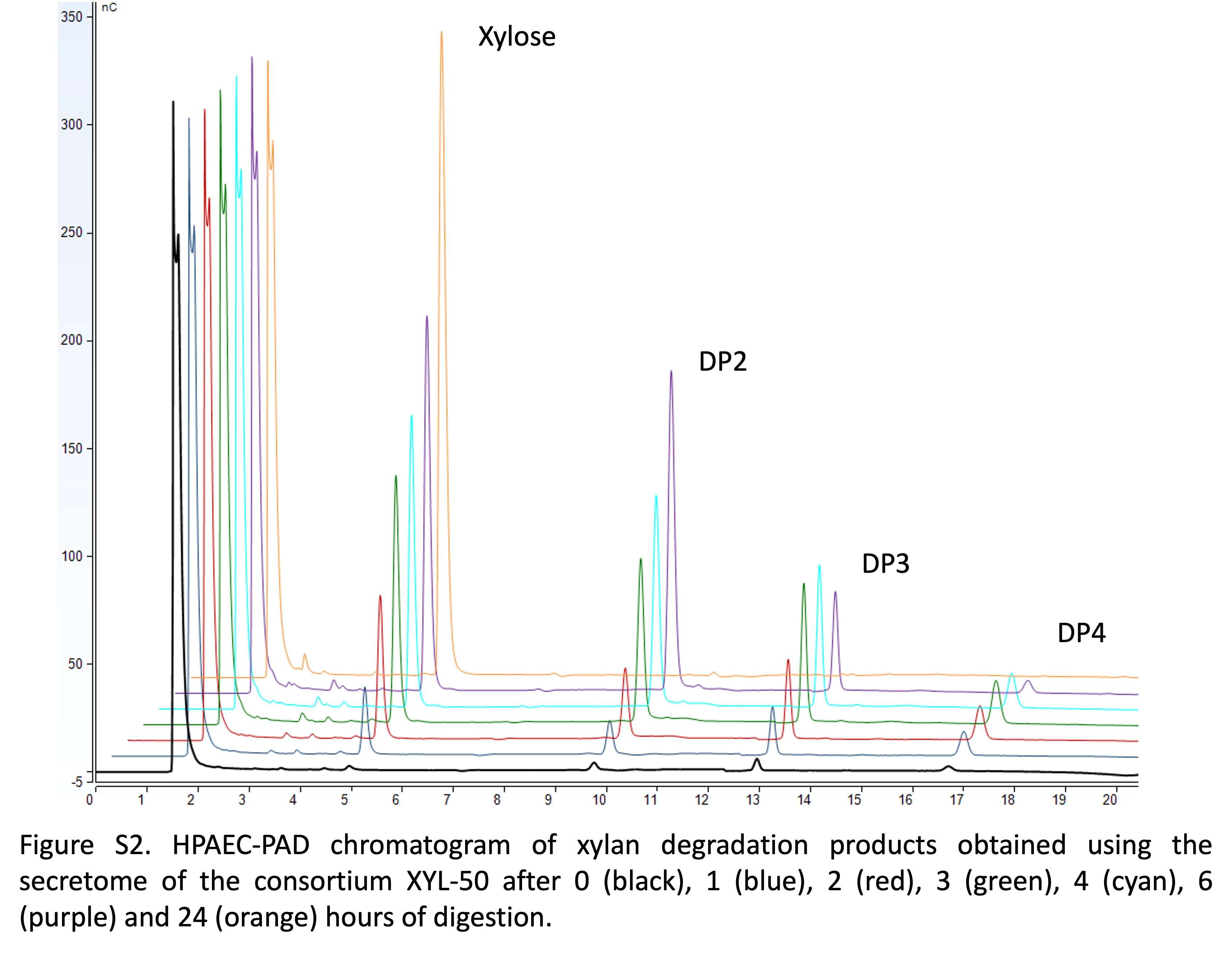

Supplement: Supplementary file 1 [file ijms-25-09887-s001.zip › Figure S2.png]

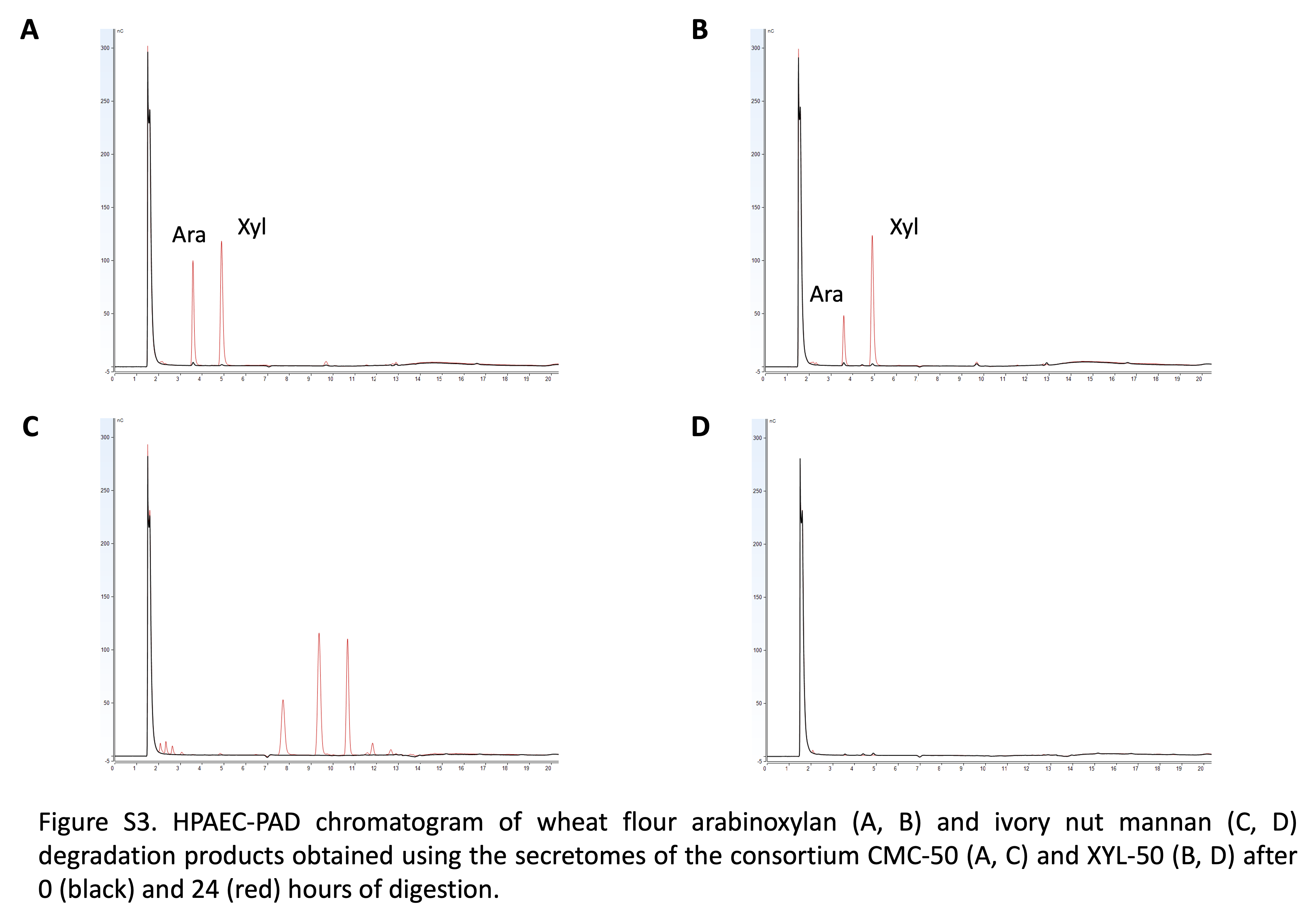

Supplement: Supplementary file 1 [file ijms-25-09887-s001.zip › Figure S3.png]

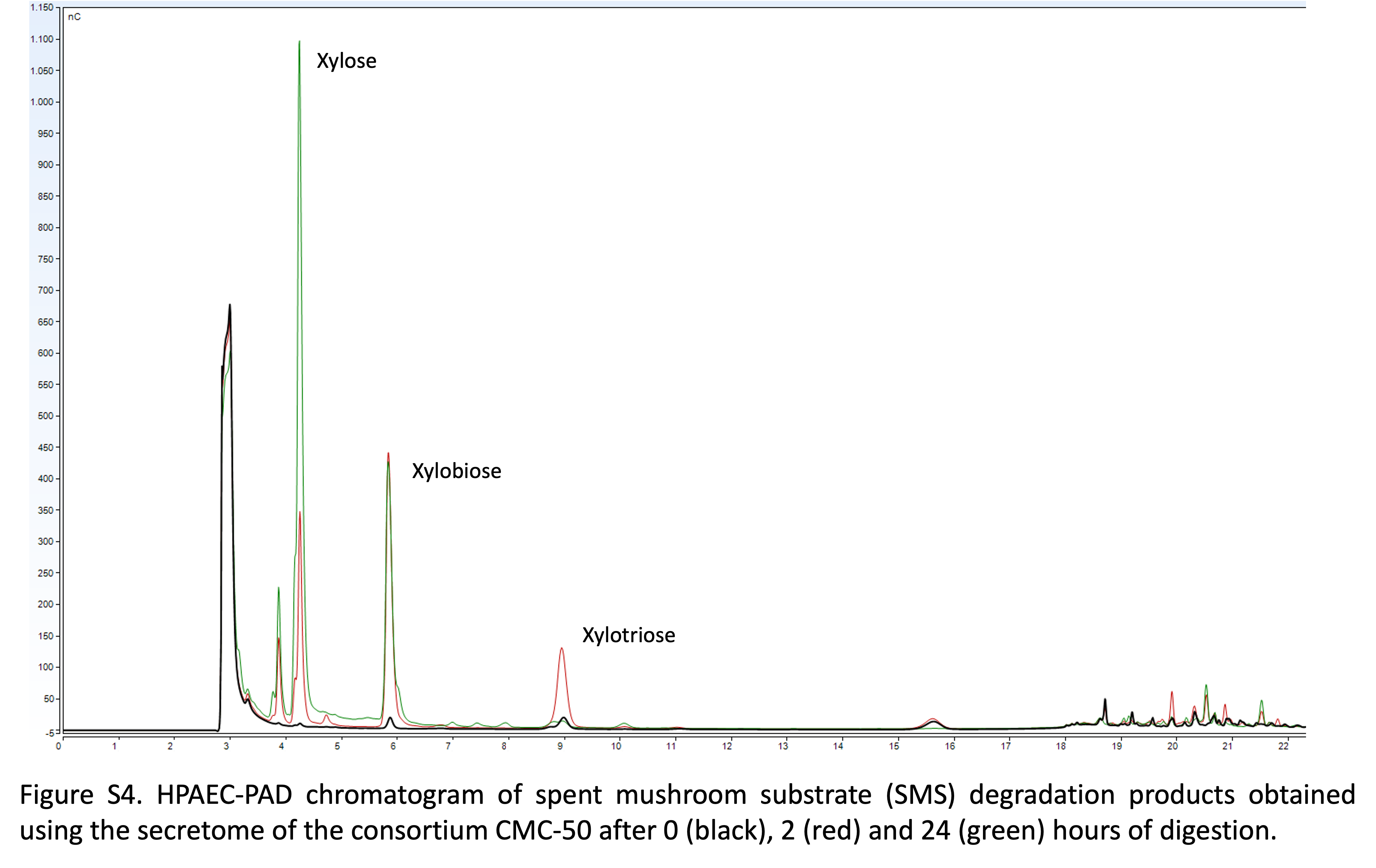

Supplement: Supplementary file 1 [file ijms-25-09887-s001.zip › Figure S4.png]

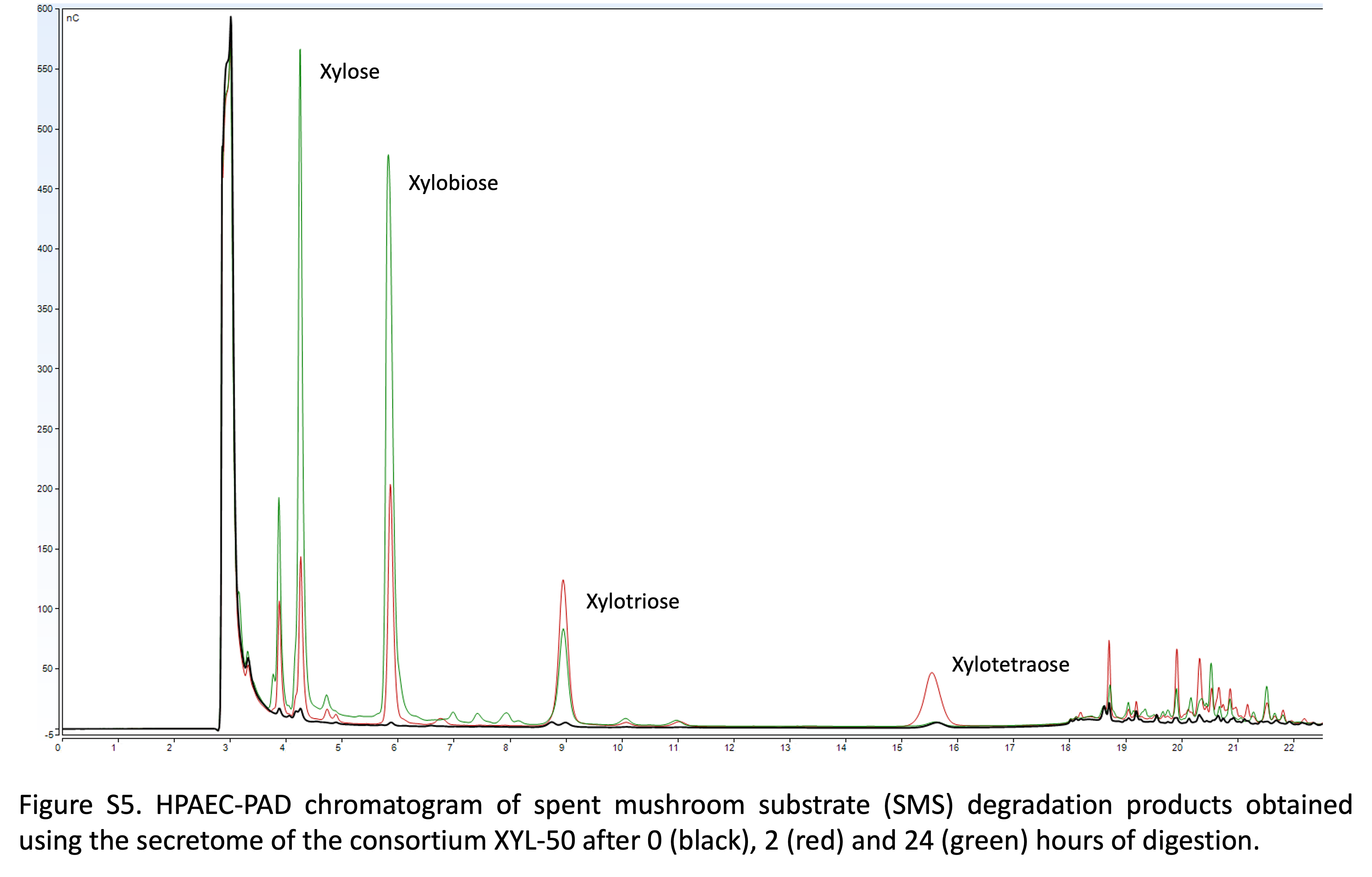

Supplement: Supplementary file 1 [file ijms-25-09887-s001.zip › Figure S5.png]
